# Prescription Practice for Diabetes Management among a Female Population in Primary Health Care

**DOI:** 10.1155/2014/103853

**Published:** 2014-03-20

**Authors:** Fouzia A. ALHreashy, Abdulelah F. Mobierek

**Affiliations:** ^1^Medical College, AL Imam Mohammed Islamic University, Riyadh, Saudi Arabia; ^2^King Fahad Cardiac Center, King Khalid Hospital, Medical College, King Saud University, P.O. Box 93254 Riyadh, Saudi Arabia

## Abstract

*Introduction.* Prescription for diabetes care is an important practice in primary care. *Methods.* This is a descriptive study carried out on at primary care clinics over a five-month period at Al Imam Medical Complex, Riyadh, Saudi Arabia. It was cross-sectional study of 160 female diabetic patients, who visited the services between January and May, 2012. Data were collected from the medical records on the clinical characteristics and drugs prescribed for their diabetic management. *Results.* The majority of the sample population (82%) was older than 40 years old. Half of them had concomitant hypertension, hyperlipidemia, and obesity. There were 500 prescriptions for diabetes management. More than 57% of participants were on two or more drugs for hyperglycemia. Metformin was the most common drug prescribed. Metformin and sulphonylurea were the most common combined medications. Most of cases ( 70%) were on a combination of antihypertensive drugs. ACE or ARBs and diuretic was the most common combined prescriptions. Statins and aspirin were used by 41% and 23.8% of the research population, respectively. *Conclusion.* Polypharmacy is a feature in diabetes care. Most of the prescription practice for diabetic care follows the recommended guidelines for hyperglycemia and hypertension. Management of dyslipidemia among diabetic patients, however, is an area that needs to be developed.

## 1. Introduction

Diabetes mellitus is considered an epidemic disease in the gulf countries. Its prevalence in Saudi Arabia is considered to be the highest in the world affecting 23–30% of adults above 30 years of age [1–3]. Diabetes mellitus is the most common endocrine problem encountered in family medicine practice [4]. Most Saudi citizens have direct access to primary care and most chronic diseases, including diabetes mellitus, are managed at this level [4]. The cost of prescriptions has been estimated to account for 30–40% of the direct cost of diabetes. A substantial increase in the cost of medication for diabetes is observed with the increased use of new antidiabetic and cardiovascular medications and use of the evidence based guidelines [5–8].

Al Maatouq Manual of Diabetes Practice indicates that the therapeutic regimen for hyperglycemia in type 2 diabetes includes the oral antidiabetic agents, insulin, or a combination of both. Metformin is the initial recommended drug for type 2 diabetes [9–12]. New drugs for hyperglycemia, such as incretins, are promising [13, 14]. Glycosylated hemoglobin A1c (HbA1c) is the test most widely used for assessment of blood glucose control. The current target level is approximately 7% with consideration of individualized care [12, 13].

Diabetes is commonly associated with risk factors for cardiovascular disease (CVD). Standard diabetes care recommends drug therapy for control of blood pressure, dyslipidemia, and assessment for the need for antiplatelets therapy [12, 15–18]. Hypertension affects 60% of the population with type 2 diabetes. The high prevalence of undiagnosed nephropathy may still favor recommendations for the use of inhibitors of the renin-angiotensin system (RAS) as first-line hypertension therapy in people with diabetes [16].

Dyslipidemia is highly prevalent among the diabetic population [18].

Low density lipoproteins (LDL) cholesterol-targeted statin therapy remains the preferred strategy for this condition [8]. Antiplatelet therapy for patients with previous CVD is highly recommended for secondary prevention. The current recommendation for primary prevention of CVD has been changed to be selective to women above 60 years of age with one additional risk factor [12, 19].

The objective of this study was to investigate the prescription practice for diabetic management of a female Saudi population and to evaluate the utilization of the medications in relation to the international guidelines.

## 2. Methodology

This study is a cross-sectional investigation of female patients with diabetes seen in the Al Imam Medical Complex from January to May 2012. The complex is an ambulatory care service belonging to the medical college at Al Imam Mohammed Islamic University in Riyadh, Saudi Arabia. It serves the students and the staff of the university and their dependents. It is a multispecialty center attached to the Ministry of Health Hospitals. It has two sections based on gender for both the staff and the clients.

According to the pharmacy registry, there are approximately 350 women with diabetes at this centre. The female cases are followed up within the primary care and family medicine clinics. The medical center has most of the facilities needed for outpatient diabetes management including manpower, basic laboratory tests, and drugs.

Consecutive samples of 160 cases who were on antidiabetic drugs and visited the center during the period of the study were included. Patients who do not have diabetes and taking metformin, such as cases with polycystic ovary syndrome or obesity, were excluded. A data collection form was designed for the purpose of the study. It contained information on the patient's demographics, smoking history, clinical data, the comorbidity, current drug history related to diabetes care, and recent laboratory data. The data were collected by reviewing the last visit in the medical records during the period of the study. The subject's drug history was obtained from the medical records and chronic drug registry of the pharmacy.

The data was analyzed using a statistical package for social science, SPSS version 20. The frequency and percentage were estimated for all variables including age, history of hypertension, hyperlipidemia, smoking, and diabetic complications. Blood pressure, body mass index (BMI) values, drug or drug class names in the last visit, HbA1c in the last six months, lipid profile, creatinine level, and urine test for albuminuria in the last year were recorded in the form. Subclassification for the data categories including the age group every 10 years, uncontrolled systolic and diastolic blood pressure, BMI classes, HbA1c above 8%, and high lipid profile levels per diabetes care standard was done. The mean and standard deviation were calculated for numerical variables. Chi-standard test was calculated for the prescription practice and *P* value ≤0.05 was considered significant. The missing data was analyzed with consideration in the percentages and the discussion.

## 3. Results

The study enrolled 160 women with diabetes. Their clinical profiles are presented in Table 1. The mean (standard deviation (SD)) age of the subjects was 53.7 (11.76) years. The majority of the population (82.4%) were older than 40 years and most (82.5%) were Saudi. There was a lack of documentation of smoking history for most of the subjects (76.3%).

A considerable proportion of cases had comorbidity including hypertension (49.4%), hyperlipidemia (45.0%), and thyroid disorders (14.4%). Less than 5% of the subjects had documented diabetic complications in their medical records.

All the subjects had a blood pressure measurement on their last visit. Mean (SD) systolic and diastolic blood pressure were 118.4 (37.9) mmHg and 69.8 (22.7) mmHg, respectively. Forty percent of cases had blood pressure readings above the recommended level (130 mmHg systolic and 80 mmHg diastolic). This percent dropped to 33% when considering the recent change in target blood pressure (140 mmHg systolic). BMI was reported for only 63.1% the subjects (101 of the 160 participants), and the mean (SD) was 33.7 (5.7) kg/m². Obesity was seen in 80.0% of the cases for whom BMI was available in the medical record.

**Figure 1:**
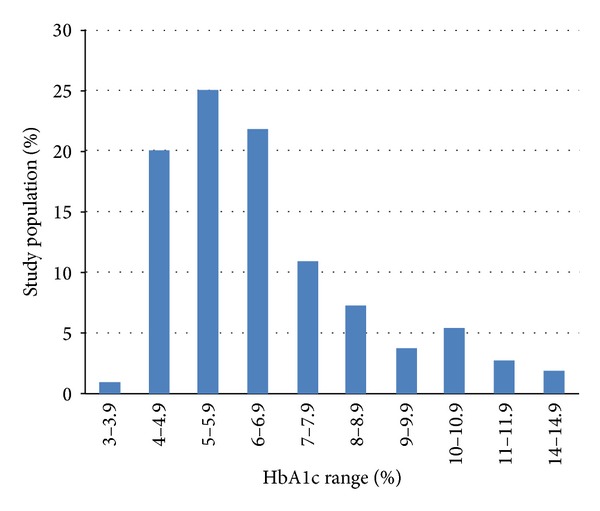
Range of HbA1c for the study population (*n* = 110).

HbA1c testing was found among 69% of the studied population with a mean (SD) value of 6.6% (2.1).  Figure 1 shows the distribution of HbA1c in subjects and indicates that most of the subjects had good glycemic control. The lipid profile was available for two-thirds of the population for total cholesterol and triglyceride levels in the last year. LDL tests were not available for 42% of the samples. The mean (SD) for the lipid profile was cholesterol 4.8 (0.9) mmol/l, triglyceride 1.7 (1.3) mmol/l, LDL 3.1 (1.3) mmol/l, and HDL 1.2 (0.3) mmol/l. Significant proportions of patients had suboptimally treated dyslipidemia based on the recommended guidelines [12]. Many medical records lacked the creatinine test (56%) or assessment of proteinuria (76%) in the last year. The majority of cases who were assessed for nephropathy had normal results.

Table 2 shows the pattern of prescriptions for diabetes management. A total of 500 prescriptions were utilized for hyperglycemia, hypertension, hyperlipidemia, and prevention of cardiovascular events for all the subjects. The majority of cases (61%) had three or more prescriptions for diabetes and cardiovascular management where the mean (SD) for the number of prescriptions per subject was 3.0 (1.5). A single medication per subject was observed in only 16% of patients.

Four drug groups were found to be commonly utilized for hyperglycemia, with metformin being the most common medication. More than half of the cases (57%) were on two drugs or more for hyperglycemia. Metformin, plus sulphonylurea, was used in 67 cases (42%) and represented the most common combination. Metformin, plus sulfonylurea, plus pioglitazone was the next most common combination and was seen in 14 cases (8.7%). Insulin monotherapy was used in 9 cases. Compared to monotherapy for treatment of hyperglycemia, combination therapy was significantly associated with the older age group, the presence of diabetes complications and health education visits, and higher HbA1c levels (Table 3).

Four drug groups were found to be commonly utilized for hypertension management. Angiotensin converting enzyme (ACE) inhibitors was the most commonly used antihypertensive agent, followed by angiotensin receptor blockers (ARBs). Most of the subjects (70%) were on combination of two or more drugs. Diuretics, including indapamide or thiazide, were commonly used for combination therapy with equal preference. *β*-Blockers were utilized in diabetes as monotherapy in seven cases. Calcium channel blockers were the least prescribed medication. The association of antihypertensive drugs with clinical characteristics was not significant (*P* ≥ 0.05).

The main drug groups utilized for hyperlipidemia were statins which were significantly associated with the older age group (*P* < 0.01) and highly significantly associated with higher BMI (*P* < 0.006). The main drug utilized as an antiplatelet agent was aspirin. The majority of aspirin users were above 50 years of age (*P* < 0.001), were hypertensive (*P* < 0.015), and had diabetic complications (*P* < 0.014). On the other hand, the aspirin use was not significant among the cases with dyslipidemia (*P* < 0.26).

## 4. Discussion

The objective of this investigation was to study the prescription practice among female patients with diabetes at the Al Imam Medical Complex. This issue is strongly related to the assessment of risk factors among the patients. Therefore, this study also gives a view on the practice of diabetes care within the primary care.

There was a high prevalence of hypertension and dyslipidemia in our study group that was similar to other gulf countries [15]. On the other hand, diabetic complications were low among the study population. Recording of whether the women in this study were smokers seemed to be neglected or not documented in their medical histories. It should be kept in mind that the population we studied is from an Islamic culture where smoking is forbidden to all. However, the prevalence of smoking among Saudis female population has been reported to range from 1 to 16% [20]. Patient's medical histories must be accurate and, in this case, cannot assume that women are nonsmokers, particularly with respect to providing proper diabetes management [21].

Blood pressure was documented for all cases as per international guidelines and is consistent with other published studies [22–24]. On the other hand, BMI was only documented in 60% of cases which is consistent with other studies [22, 25, 26].

Weight measurement is recommended as part of diabetes management and needs to be consistently recorded as part of routine nursing practice. Obesity is a coexisting risk factor for patients with diabetes and found to be highly prevalent in subjects in many studies [2, 22, 25]. Insulin and pioglitazone therapies were used in 28.4% of the population and may have contributed to weight gain in patients taking these medications.

The laboratory tests for assessment of HbA1c, kidney function, and lipid profile were underutilized compared to the recommended guidelines and other studies and similar to a Malaysian study [20, 24, 26]. This may be related to lack of training of primary care doctors, shortages in the availability of tests, and shared care of the patients with other medical centers. The level of diabetic control among cases with available HbA1c tests (69%) revealed good control among most of them. By comparison, it is similar to that reported for the Canadian population [22, 27] and better than that of general Middle East population where the mean HbA1c is 8-9% [28]. Overreduction of HbA1c was observed in this data which may be due to management of hyperglycemia based on spot blood glucose testing. Several possible reasons may account for the good control, including the high level of education of this population, ease of access to health care, shared care with different services, and the availability of most drugs needed for diabetes care. Furthermore, for a prescription to be refilled every two months, patients must see a physician, thereby improving the opportunity for patient care.

The commonly used medications for hyperglycemia control were limited to the available drugs in the services. Metformin was the most commonly prescribed medication for control of hyperglycemia and this follows the international guidelines. This is in contrast to the Middle East data where metformin appears to be less frequently used to initiate antidiabetic therapy [5, 28]. Despite knowing the benefits of metformin, approximately 13% of the cases in our study are not being treated with it. The side effects of metformin or the presence of type 1 diabetes may explain these findings. On the other hand, insulin therapy seems to be underutilized in our study and is consistent with previous studies [8, 29, 30]. Our study was conducted at an ambulatory care facility where most of the cases were not complicated and physicians' experience with using insulin may not be extensive.

Combination therapy for controlling hyperglycemia seems to be used more among the older age group, in more complicated cases [31] and in patients with higher HbA1c values. There was also marked underutilization of dipeptidyl-4 inhibitors and alpha glucoside inhibitors, mostly due to unavailability of these medications in the formulary of the complex at the time of the study.

Efforts to control for blood pressure to the recommended level of 130 mmHg, systolic, and 80 mmHg, diastolic, have been achievable in only about one-third of the patients, despite the availability of varieties of antihypertensive drugs within the center. This inability to reduce blood pressure has also been observed in other institutes [27, 32]. The intensive control of blood pressure is still a debate in the literature as its benefit is small in the reduction of stroke compared to standard targets. Furthermore, the standards of diabetic care have raised the target systolic blood pressure from 130 mmHg to 140  mmHg [12, 27, 33]. The utilization of antihypertensive medications showed that most of subjects were on either ACE inhibitor or ARBs. No patients were on both medications as per SHMS guidelines [23]. The use of other classes of antihypertensive drugs in some subjects as monotherapy is probably due to a recent diagnosis of diabetes in addition to established hypertension, or coexisting problems of palpitation or anxiety symptoms, presence of coronary artery disease, or thyroid disorder. Thiazide is among the recommended diuretic combination therapy, yet the diuretics (indapamide or thiazide) were chosen with equal preference [33]. The availability of combined antihypertensive preparations (thiazide and ARBs in one tablet) at the center may explain the preference of diuretic compared to the low use of calcium channel blockers. Surprisingly, there was not a significant association between the clinical characteristics with monotherapy versus combination therapy among the samples. Further study with bigger sample size to test this association is needed.

The prescription practice of our sample population showed underutilization of the complete lipid profile test of diabetes management, particularly LDL. This practice can be accepted for cases with a previous cardiovascular event provided that they will receive statin therapy [12]. However, the underutilization of antilipid drugs (statins and fibrates) is marked in these patients despite their availability. Reducing LDL below a target level of 2.6 mmol/l is still difficult to attain in many populations including the US [8, 34]. The positive association of statin use with older age group in our study correlated positively with the current guidelines. These findings, together with high prevalence of obesity, stress the need for a clinical dietician in the complex [12].

Prescription of aspirin was seen at an earlier recommended age, that is, 50 years. This may be because of an increased awareness of the benefits of aspirin and following the old recommendation for the use of aspirin in the primary prevention of CVD [32, 35]. Interestingly, the lack of association of aspirin use among cases with dyslipidemia adds further need for proper management of dyslipidemia among diabetes management. Consistent with other studies, the primary prevention of CVD among type 2 diabetes patients is still an area of development [19].

Other areas that we found to be underutilized were fundus examinations and the health education clinic at the Al Imam Medical Complex. However, this result should be cautiously regarded since these patients are under shared care with other medical centers.

## 5. Conclusion

This study investigated the prescription practice for a subpopulation of female patients with diabetes at a primary care center in Saudi Arabia. Our study revealed that the prescription practice for these female patients is consistent with standard diabetes care and SHMS guidelines. However, prescriptions for dyslipidemia still appear to be an area of attention in primary care. The utilization of insulin and incretins was expected to be higher among type 2 uncontrolled cases. This study had a small sample size and extrapolation of our results is limited. More importantly, this study sheds light on the trend of prescriptions practice for diabetes care and will help to draw attention, at the national level, for comprehensive diabetes care management with gender differences.

## Figures and Tables

**Table 1:** Clinical characteristics of the study population (*n* = 160).

Category	Value (%)
Age distribution, in years	
11–20	1 (0.6)
21–30	2 (1.3)
31–40	16 (10.0)
41–50	34 (21.3)
51–60	66 (41.3)
>60	31 (19.4)
Nationality	
Saudi	133 (83.1)
Non-Saudi	27 (16.9)
Smoking history	
Unknown	122 (76.3)
Not smoking	32 (23.8)
Comorbid conditions	
Hypertension	79 (49.4)
Dyslipidemia	72 (45.0)
Thyroid disorder	23 (14.4)
Diabetic complications	
Retinopathy	9 (5.6)
Neuropathy	6 (3.8)
Stroke	3 (1.9)
Ischemic heart disease	1 (0.6)
Blood pressure (mmHg)	
Systolic BP > 130	56 (35.0)
Systolic BP ≥ 140	39 (24.4)
Diastolic BP > 80	33 (20.6)
BMI (value), kg/m^2^	
Normal (18–25)	3 (3)
Overweight (25.1–29.9)	17 (16.8)
Mild obesity (30–34.9)	33 (32.6)
Moderate obesity (35–39.9)	40 (40)
Morbid obesity (≥40)	8 (8)
Fundus examination	49 (30.9)
Health education	48 (30.0)
Laboratory results (*n* = number of participants that had the test)	
HBA1c >8.0 (*n* = 110)	23 (20.9)
Serum creatinine >135 umol/L (*n* = 70)	None
Albuminuria positive (*n* = 38)	5 (13.2)
Cholesterol ≥ 5.2 mmol/L (*n* = 121)	37 (30.5)
LDL ≥ 2.6 mmol/L (*n* = 94)	65 (69.2)
HDL ≤ 1.3 mmol/L (*n* = 90)	58 (64.4)
Triglyceride ≥ 1.7 mmol/L (*n* = 118)	14 (11.9)

**Table 2:** Distribution of prescriptions for diabetes and cardiovascular management among the total study population.

Drug group according to clinical problem	Drug name	Value (%) (regardless of combination status)	Value (%) monotherapy
Drugs for hyperglycemia	Metformin	139 (86.9)	55 (39.6)
	Sulphonylurea	77 (48.1)	6 (7.8)
	Pioglitazone	23 (14.4)	0
	Insulin	23 (14.4)	9 (39.1)
	Others (acarbose, sitaglibtin)	5 (3.1)	0
	Total prescriptions	267	70

Antihypertensive drugs	ACE inhibitors and ARBs	61 (38.1)	38 (23.8)
	Diuretics (thiazide + indapamide)	26 (16.2)	5 (3.1)
	B-blocker	21 (13.1)	7 (33.3)
	Calcium channel blocker	8 (5)	1 (12.5)
	Total prescriptions	124	49

Lipid lowering agents	Statin	66 (41.3)	64
	Others (fibrate, ezetimibe)	2	0
	Total prescription	68	64

Antiplatelets therapy	Aspirin	38 (23.8)	35
	clopidogrel	3	0
	Total prescriptions	41	35

**Table 3:** Association of clinical characteristics with antidiabetic medications.

Category	Antidiabetics		
	On monotherapy	On combination*	*P* value
Number of patients (*n* = 155)	69	86	
Age in years, mean (SD)	50.8 (12.4)	55.6 (11.1)	0.020
Systolic BP mmHg, mean (SD)	116.9 (40.4)	118.5 (36.4)	0.793
Diastolic BP mmHg, mean (SD)	69.3 (22.8)	69.7 (23.1)	0.911
BMI, mean (SD)	33.2 (4.4)	34.0 (6.5)	0.480
Hyperlipidemia, *n* value (%)	28 (40.6)	40 (46.5)	0.516
Hypertension, *n* (%)	38 (55.1)	41 (47.7)	0.420
Thyroid disorder, *n* value (%)	12 (17.4)	10 (11.6)	0.358
Diabetic complications^Ω^	5 (7.2)	16 (18.6)	0.040
HbA1c, mean (SD)	5.9 ± 1.8	7.1 ± 2.2	0.006
Cholesterol in mmol/L, mean (SD)	4.9 ± 0.8	4.7 ± 1.1	0.480
Triglyceride in mmol/L, mean (SD)	1.4 ± 0.8	1.9 ± 1.5	0.161
HDL in mmol/L, mean (SD)	1.3 ± 0.3	1.2 ± 0.3	0.857
LDL in mmol/L, mean (SD)	3.4 ± 1.8	2.9 ± 0.9	0.096
With health education visit, *n* value (%)	14 (20.3)	31 (36.0)	0.034
With patient on antihypertensive drugs	19 (27.5)	31 (36.0)	0.100
With patient on antilipid drugs	26 (37.7)	39 (45.3)	0.413
With patient on antiplatelet	14 (20.3)	24 (27.9)	0.348

*Combination of four drugs: metformin + sulphonylure + actos + insulin.

^Ω^Diabetic complications: neuropathy + retinopathy + stroke + IHD + proteinuria. IHD: ischmic hear disease.
